# The effect of prolonged of warm ischaemic injury on renal function in an experimental ex vivo normothermic perfusion system

**DOI:** 10.1186/s12967-015-0571-4

**Published:** 2015-06-30

**Authors:** Sarah A Hosgood, K Shah, M Patel, M L Nicholson

**Affiliations:** Department of Infection, Immunity and Inflammation, University of Leicester, Leicester, UK; Department of Surgery, University of Cambridge, NIHR Cambridge Biomedical Research Centre, Cambridge, CB2 0QQ UK

**Keywords:** Kidney, Transplantation, Warm ischaemia, Reperfusion

## Abstract

**Background:**

Donation after circulatory death (DCD) kidney transplants inevitably sustain a degree of warm ischaemic injury, which is manifested clinically as delayed graft function. The aim of this study was to define the effects of prolonged periods of warm ischaemic injury on renal function in a normothermic haemoperfused kidney model.

**Methods:**

Porcine kidneys were subjected to 15, 60, 90 (n = 6 per group) and 120 min (n = 4) of in situ warm ischaemia (WI) and then retrieved, flushed with cold preservation fluid and stored in ice for 2 h. Kidneys then underwent 3 h of normothermic reperfusion with a whole blood-based perfusate using an ex vivo circuit developed from clinical grade cardiopulmonary bypass technology.

**Results:**

Creatinine clearance, urine output and fractional excretion of sodium deteriorated sequentially with increasing warm time. Renal function was severely compromised after 90 or 120 min of WI but haemodynamic, metabolic and histological parameters demonstrated the viability of kidneys subjected to prolonged warm ischaemia.

**Conclusions:**

Isolated kidney perfusion using a warm, oxygenated, red cell-based perfusate allows an accurate ex vivo assessment of the potential for recovery from warm ischaemic injury. Prolonged renal warm ischaemic injury caused a severe decrement in renal function but was not associated with tissue necrosis.

## Background

The continuing shortage of suitable transplant kidneys has led to an increasing reliance on donation after circulatory death (DCD) donors [1, 2]. The challenge for the future is to achieve a significant increase in transplant activity by using increasingly marginal donors without compromising success rates. In the UK the number of DCD renal transplants has plateaued in recent years [3]. The vast majority of these DCD kidneys are from controlled donors and uncontrolled donors have remained a relatively untapped additional potential source of donor organs. Uncontrolled DCD kidneys are exposed to more prolonged warm ischaemic injury and this is reflected in high rates of primary non function and delayed graft function [4, 5].

Previous work by our group investigated the relationship between warm ischaemia, with a duration of 40 min or less, and the severity of renal ischaemia reperfusion injury in an ex vivo porcine model of controlled kidney DCD [6]. There was a clear gradation between warm time and markers of oxidative injury. Increasing warm injury was also closely correlated with the subsequent decrement in renal function. Further work is required to define the relationship between periods of more prolonged warm ischaemic injury and the development of irreversible renal injury.

The aim of this study was to assess the effects of prolonged warm ischaemia (WI) on renal function using an isolated haemoperfused porcine kidney model.

## Methods

Landrace female pigs weighing approximately 50 kg were terminated under schedule 1 of the Home Office Scientific act 1986 regulations. After exsanguination and collection of blood into a sterile receiver containing 25,000 units of heparin the kidneys were exposed and mobilized. Kidneys were randomly assigned into 1 of 4 groups and either subjected to 15 min (n = 6), 60 min (n = 6), 90 min (n = 6) or 120 min (n = 4) of warm ischaemia (WI) before being flushed with 500 ml of Soltran at 4°C. Kidneys were then stored in ice for 2 h.

### Reperfusion

After preservation kidneys were reperfused for 3 h on an ex vivo kidney perfusion system as previously described [6]. In brief, the system was based on paediatric cardiopulmonary bypass technology incorporating a centrifugal pump, heater exchanger, oxygenator and infusion pumps. Kidneys were perfused at set mean arterial pressure (85 mmHg) for 3 h with an oxygenated autologous blood based solution at 38°C. Creatinine was added to the perfusate to achieve a concentration of 1,000 μmol/L for measurement of creatinine clearance.

Plasma and urine samples were collected at hourly time points during reperfusion.

### Outcome measures

The renal blood flow (RBF), mean arterial pressure (MAP), urine output (UO) and temperature were recorded continuously. Intra-renal resistance (IRR) was calculated by MAP/RBF. A blood gas analyser (OPTI Medical CCA-TS) was used to record PCO_2_, PO_2_, pH, Base Excess and HCO_3_^−^ for acid–base homeostasis. Oxygen consumption was calculated by the (PO_2_ (arterial) − PO_2_ (venous) × RBF/g.

Serum and urine samples were obtained hourly for biochemical analysis and whole blood was collected pre and post ex vivo reperfusion for haematological analysis. Creatinine clearance (CrCl) was calculated by urine creatinine × urine output (ml/min)/plasma creatinine. To assess tubular injury, the fractional excretion of Na^+^ was calculated by (urine Na^+^ × Urine output (ml/min)/CrCl × plasma Na^+^). The protein/creatinine ratio was also calculated.

Levels of Neutrophil Gelatinase-Associated Lipocalin (NGAL) were quantified in urine samples collected post perfusion using a porcine NGAL enzyme-linked immunosorbent assay (BioPorto Diagnostics). Standards, diluted samples (1/5,000) and controls were added to wells pre-coated with a monoclonal antibody against porcine NGAL. The plate was incubated for 1 h at room temperature on a shaking platform (200/min). Wells were washed with buffer to remove any unbound material. Biotinylated Pig-NGAL antibody was added to each of the wells and the plate was incubated for 1 h at room temperature. Unbound detection antibody was washed away and wells were incubated with HRP-conjugated streptavidin for 1 h at room temperature on a shaking platform (200/min). A series of washing steps was performed to removed unbound conjugate. A peroxidase substrate containing tetramethylbenzidine was added to react with the HRP-conjugated streptavidin. After 10 min the reaction was stopped by adding sulphuric acid and the intensity of the yellow colour generated was read at 450 nm using a spectrometer.

### Histology

A wedge biopsy was taken after 3 h of reperfusion. The tissue was fixed in 10% formal saline for 24 h then embedded in wax. Sections were cut at 4 μm and stained with Haematoxylin and eosin (H&E). Ten fields of each section were assessed for tubular dilatation, tubular debris, epithelial flattening, interstitial oedema and glomerular shrinkage. Sections were scored 0 indicating no abnormalities, 1 mild changes, 2 moderate and 3 severe injury.

### Statistics

Data are presented as the mean ± SD. Continuous data was compared using ANOVA for parametric or the Kruskal–Wallis test with Dunn’s multiple comparison for nonparametric data. Area under the curve (AUC) was calculated for continuous data. Statistical analysis was performed using Graphpad Prism version 6 software (La Jolla, CA, 92037, USA). P < 0.05 was considered statistically significant.

## Results

### Renal haemodynamics and oxygen consumption

**Figure 1:**
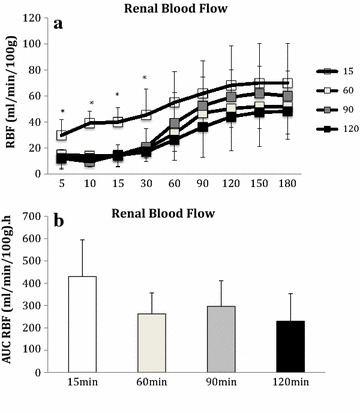
**a** Mean renal blood flow during 3 h of reperfusion. Kidneys were subjected to 15, 60, 90 and 120 min of warm ischaemia (WI) followed by 2 h of static cold storage (SCS). *P < 0.05 15 min versus 60, 90 and 120 min groups. **b** Area under the curve renal blood flow. Kidneys were subjected to 15, 60, 90 and 120 min of warm ischaemia (WI) followed by 2 h of static cold storage (SCS). P = 0.084.

Renal blood flow was significantly higher in the 15 min WI group for the first 30 min of reperfusion compared to the 60, 90 and 120 min groups (P = 0.009; Figure 1a). Thereafter, levels were similar in all groups. There was no significant difference in the area under the curve (AUC) of renal blood flow between the groups (P = 0.085; Figure 1b). Overall, the AUC intra-renal resistance was significantly lower in the 15 min WI group (AUC 15 min 5.7 ± 2.7, 60 min 9.4 ± 5.1, 90 min 13.3 ± 5.8 and 120 min 14.4 ± 6.0 ml/mmHg, 100 g h; P = 0.040).

There was no significant difference in levels of oxygen consumption after 1 or 3 h of reperfusion between the groups (P = 0.243, 0.381; Table 1). There was a numerical rise in the levels during reperfusion in all groups but this did not reach statistical significance.

**Table 1:** Levels of oxygen consumption at 1 and 3 h or reperfusion

Oxygen consumption (ml/min/g)	1 h	3 h	P value
15 min	33.9 ± 16.1	43.9 ± 17.1	0.320
60 min	19.5 ± 7.5	31.3 ± 17.1	0.080
90 min	25.2 ± 13.5	41.1 ± 12.9	0.063
120 min	20.2 ± 12.1	31.1 ± 17.2	0.340

Values are mean ± SD.

### Renal function

**Figure 2:**
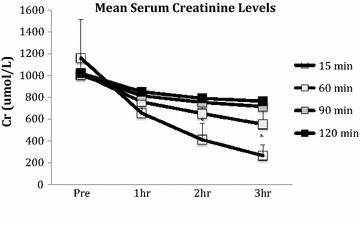
Mean serum creatinine levels during 3 h. Kidneys were subjected to 15, 60, 90 and 120 min of warm ischaemia (WI) followed by 2 h of static cold storage (SCS). *P = 0.004, 0.001, 15 min versus 90 and 120 min groups at 2 and 3 h of reperfusion, respectively.

The level of serum creatinine fell in all groups. Levels were significantly lower at 2 and 3 h reperfusion in the 15 min WI group compared to the 90 and 120 min WI groups (P = 0.004, 0.001; Figure 2). There was no significant difference in the AUC serum creatinine (15 min 1790 ± 566, 60 min 2203 ± 384, 90 min 2434 ± 132 and 120 min 2541 ± 84 μmol/L h; P = 0.081).

**Figure 3:**
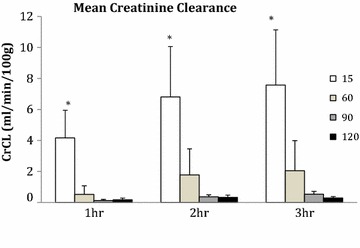
Mean creatinine clearance at 1, 2 and 3 h of reperfusion. Kidneys were subjected to 15, 60, 90 and 120 min of warm ischaemia (WI) followed by 2 h of static cold storage (SCS). *P < 0.05 15 min versus 90 and 120 min.

Creatinine clearance increased during reperfusion in the 15 and 60 min WI groups but remained low and constant in the 90 and 120 min WI groups. Levels were significantly higher in the 15 min WI group compared to the 90 and 120 min WI groups at each of the hourly time points (P = 0.002, 0.004, 0.003; Figure 3). The AUC of creatinine clearance was significantly higher in the 15 min WI group compared to the 90 and 120 min groups (AUC 15 min 12.7 ± 5.5, 60 min 3.1 ± 2.9, 90 min 0.70 ± 0.20 and 120 min 0.56 ± 0.20 ml/min/100 g h; P = 0.002).

**Figure 4:**
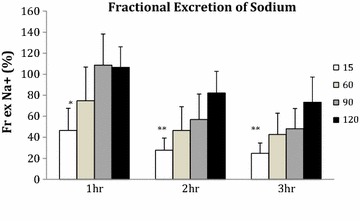
Mean fractional excretion of sodium at 1, 2 and 3 h of reperfusion. Kidneys were subjected to 15, 60, 90 and 120 min of warm ischaemia (WI) followed by 2 h of static cold storage (SCS). * P = 0.003 15 min versus 90 and 120 min. **P = 0.005, 0.007 15 min versus 120 min group, respectively.

Tubular function improved in all groups but remained significantly better in the 15 min WI group compared to the 90 and 120 min WI groups at 1 h (P = 0.003) and the 120 min WI group at 2 and 3 h of reperfusion (P = 0.005, 0.007; Figure 4). AUC levels of fractional excretion of sodium were significantly lower in the 15 min WI group compared to the 90 and 120 min WI groups (AUC 15 min 63.4 ± 25.4, 60 min 105.3 ± 48.1, 90 min 135.3 ± 43.8, 120 min 172.2 ± 41.1% h; P = 0.004).

**Figure 5:**
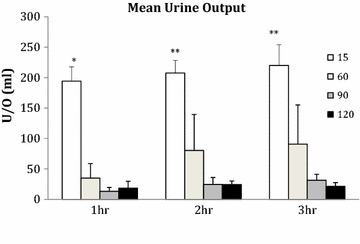
Mean urine output at 1, 2 and 3 h or reperfusion. Kidneys were subjected to 15, 60, 90 and 120 min of warm ischaemia (WI) followed by 2 h of static cold storage (SCS). *P < 0.001 15 min versus 60, 90 and 120 min. **P = 0.002, 0.001 15 min versus 90 and 120 min, respectively.

In the first hour, the urine output was significantly higher in the 15 min WI group compared to the 60, 90 and 120 min WI groups (P < 0.0001). The amount of urine production increased throughout reperfusion in the 15 and 60 min WI groups. At 2 and 3 h of reperfusion it was significantly higher in the 15 min WI group compared to the 90 and 120 min WI groups (P = 0.002, 0.001; Figure 5). The total urine output during 3 h of reperfusion was significantly higher in the 15 min WI (622 ± 40 ml) group compared to the 90 (69 ± 17 ml) and 120 min WI (64 ± 14 ml) groups (P = 0.001). The total amount of urine produced was 206 ± 142 ml in the 60 min WI group.

### Injury biomarkers

Levels of NGAL in the urine after 3 h of reperfusion were significantly lower in the 15 min WI group (15.3 ± 21.5 pg/ml) compared to the 60 (102.6 ± 21.2 pg/ml) and 90 min (63.8 ± 71.0 pg/ml) WI groups (P = 0.014). The protein/creatinine ratio (P/Cr) was significantly higher in the 90 and 120 min WI groups at 1 h compared to the 15 min WI group (P = 0.007; Table 2). Levels remained low in the 15 min WI group at 2 and 3 h of reperfusion. In the 90 and 120 min WI groups the P/Cr ratio fell significantly by 3 h (P = 0.002, 0.042; Table 2). Levels were numerically lower in the 60 min WI group. After 3 h of reperfusion the P/Cr ratio was significantly higher in the 120 min WI group compared to the 15 min WI group (P = 0.045; Table 2).

**Table 2:** Protein/Creatinine ratio (mg/mmol/L) at 1, 2 and 3 h of reperfusion

	1 h	2 h	3 h
15 min	5.2 ± 3.4	4.3 ± 1.0	6.4 ± 1.7
60 min	12.9 ± 8.9	6.3 ± 3.6	6.3 ± 2.3
90 min	20.0 ± 8.8*	7.8 ± 6.5	6.1 ± 3.9^Ψ^
120 min	24.2 ± 10.4*	13.7 ± 6.8	12.3 ± 5.8**^Ψ^
P value	0.007	0.243	0.045

* P < 0.05 between the 15 min and 90 min and 120 min groups. ** P < 0.05 between the 15 min and 120 min groups. ^Ψ^P < 0.05 between 1 and 3 h.

### Histology

The histological evaluation after reperfusion showed a significant increase in the degree of vacuolation in the 60 and 90 min WI groups compared to the 120 min WI group (P = 0.033; Table 3). There was more glomerular shrinkage in the 90 min WI group compared to the 15 min WI group (P = 0.017; Table 3).

**Table 3:** Histological evaluation assessing tubular dilatation (Tubular Dil), tubular debris, vacuolation, interstitial oedema and glomerular shrinkage in wedge biopsies after 3 h of reperfusion

Histology	15 min	60 min	90 min	120 min
Tubular Dil	1.62 ± 0.33	1.80 ± 0.45	1.65 ± 0.43	1.40 ± 0.44
Tubular debris	1.38 ± 0.34	1.37 ± 0.37	1.80 ± 0.53	1.90 ± 0.58
Vacuolation	2.20 ± 0.10	2.45 ± 0.63*	2.37 ± 0.61*	1.20 ± 0.59
Interstitial oedema	1.35 ± 0.25	1.48 ± 0.34	1.38 ± 0.36	1.35 ± 0.78
Glomerular	1.40 ± 0.32**	1.80 ± 0.30	1.95 ± 0.23	1.58 ± 0.25

Sections were stained with H&E and scored over 10 fields, 0 indicating no abnormalities, 1 mild changes, 2 moderate and 3 severe injury.

Vacuolation (* P = 0.033, Tukey’s post test P < 0.05) 60 min WI versus 120 min WI and 90 min WI versus 120 min WI. Glomerular shrinkage (** P = 0.018, Tukey’s post test P < 0.05) 15 min WI versus 90 min WI. Score shown are mean ± SD.

All groups showed significant tubular dilatation, narrowing of the epithelium and interstitial oedema. Several of the biopsies in the 60, 90 and 120 min WI groups had hyaline casts within the tubules. There was no evidence of ischaemic tubular injury or necrosis in any of the samples.

## Discussion

This study shows that porcine kidneys subjected to prolonged warm ischaemic injury of 90–120 min have very poor early renal function after reperfusion but remain viable. This conclusion is made on the basis that these kidneys had acceptable renal blood flow that improved over the 3-h perfusion period and they also produced urine throughout reperfusion, albeit in relatively small volumes. Moreover, there was no histological evidence of ischaemic tubular injury after 3 h of reperfusion. Although there was also no evidence of necrosis in any biopsy material, 3 h of reperfusion is likely to be too short to show definitive changes. Finally, prolonged WI did not reduce renal oxygen consumption. In the clinical situation kidneys with such prolonged WI injury might be expected to have a very high rate of delayed graft function but primary non-function due to ischaemic necrosis would not be inevitable. An important caveat to these conclusions is that the available data is indirect and we accept that kidney viability could only have been assessed definitively by transplantation of the kidneys studied and this was not part of the model used here.

As expected 15 min of WI had very little, if any, adverse effect. Fifteen minutes was chosen as the minimal time under study as a reflection of the usual mean warm ischaemic time in human kidneys transplanted from controlled DCDs [7]. These kidneys had high blood flow, produced a large volume of urine and demonstrated good renal function. Kidneys in the 60 min WI group were able to maintain a low level of creatinine clearance over the 3-h reperfusion period and produced a urine output of more than 50 ml/h. This duration of warm ischaemia did not affect tissue viability.

Ex vivo normothermic perfusion using the system described here provides a straightforward, reproducible and clinically relevant method of assessing the effects of ischaemia and the potential for recovery from a particular level of insult [8, 9]. The system can also be used to re-condition injured kidneys prior to transplantation and has great potential as a platform for the delivery of pre-transplant therapies [10].

Porcine kidneys were used in this study as they provide an accurate model of the human situation largely because of their multilobular structure, which contrasts with the unilobular anatomy of rodent and dog kidneys [11]. The physiological characteristics and ischaemic tolerances of porcine kidneys are also similar to those of human kidneys [12]. Porcine kidneys are also not without limitations as they are procured from healthy adolescent pigs with no co-morbidities and this is a closer analogue of live donor kidneys rather than donation after circulatory death human kidneys.

Parekh et al. analysed the effects of warm and cold ischaemia in human kidneys in a study of 40 patients undergoing partial nephrectomy for renal tumours [13]. The renal pedicle was cross-clamped to achieve haemostatic tumour resection. This was either under warm conditions for a mean (Range) time of 32.3 (15–53) min (n = 27) or using surface hypothermia using ice slush for a mean (range) cold ischaemic period of 30 and 48 min (n = 13). Core renal biopsies were obtained before, during and 5 min after the ischaemic insult. There was a small and transient increase in serum creatinine that did not correlate with the duration of ischaemia. Renal structural and ultrastructural changes were much less severe than observed in small animal models and did not correlate with peak creatinine or duration of ischaemia. In particular, mitochondrial swelling resolved rapidly during the 5 min of reflow. We also found only mild to moderate histological changes in porcine kidneys subjected to 60 min of warm ischaemic injury. Nonetheless, the value of histological parameters is unclear as they have been shown not to correlate with early renal function after clinical transplantation [14]. Parekh et al. concluded that human kidneys can safely tolerate 30–60 min of ischaemic injury and this is consistent with the findings described here using the porcine model [13].

Our study was also limited by the absence of a clinically relevant period of storage under cold ischaemic conditions after the initial warm ischaemic insult. The cold ischaemic time of 2 h was required to transport kidneys from the animal facility to the perfusion laboratory and additional cold ischaemic was deliberately avoided as we wanted to study the effects of WI per se as far as possible. The addition of a period of 2 h cold storage to WI does not cause a further decrement in renal function during reperfusion [15]. Previous studies using this model have demonstrated that the deleterious effects of 1 min of WI are equivalent to those from a 1-h period of static cold storage and that there is a pronounced cumulative effect when porcine kidneys sustain a combination of warm and cold ischaemic injuries. In an ex vivo study of similar design, which compared the effects of 7, 15, 25 and 40 min WI on porcine renal function, there was a clearly delineated relationship between the duration of WI and progressive loss of renal function [6]. The present study also demonstrated a WI-dependent variation in renal function but there were no differences between the kidneys exposed to 90 and 120 min WI. This was a function of the fact that creatinine clearance and urine output were extremely low when WI exceeded 60 min.

Allografts from human controlled DCD and donation after brain death donors have similar rates of delayed graft function but those from uncontrolled DCDs, which have much more prolonged WI, have much higher rates of DGF [16]. It has proved more difficult to define the WI limits in human renal transplantation. Whilst some studies find that long WI is associated with higher rates of primary non-function, others were unable to confirm this [17–20]. Approximately 8% of donation after brain death (DBD) and 18% of DCD donor kidneys are retrieved but then declined for transplantation [1]. DCD kidneys are commonly declined if the donor has suffered a significant period of cardiorespiratory arrest because of concern about the duration and severity of the WI insult [21]. Ex vivo assessment during normothermic perfusion is likely to give additional information that would increase the likelihood of warm ischaemically injured kidneys being accepted for transplantation.

Isolated kidney perfusion using a warm, oxygenated, blood-based perfusate allows recovery of function ex vivo and this provides the basis for an accurate assessment of the severity of WI injury. In turn, this may be helpful in assessing the potential for recovery from prolonged ischaemic insults.

## Conclusion

In this study prolonged renal WI injury caused a severe decrement in renal function but was not associated with tissue necrosis, suggesting that large animal kidneys can tolerate more ischaemia than previously realised. This also suggests that human kidneys may also tolerate longer durations of ischaemia which may help to increase the number of available kidneys for transplantation.

## References

[1] Johnson RJ, Bradbury LL, Martin K, Neuberger J, UK Transplant Registry (2004) Organ donation and transplantation in the UK-the last decade: a report from the UK national transplant registry. Transplantation 97(Suppl 1):S1–S27 10.1097/01.TP.0000438215.16737.68 24356460

[2] Summers DM, Johnson RJ, Allen J, Fuggle SV, Collett D, Watson CJ (2010). Analysis of factors that affect outcome after transplantation of kidneys donated after cardiac death in the UK: a cohort study. Lancet.

[3] https://nhsbtmediaservices.blob.core.windows.net/organ-donationassets/pdfs/activity_report_2013_14.pdf. Accessed 15 May 2015

[4] Barlow AD, Metcalfe MS, Johari Y, Elwell R, Veitch PS, Nicholson ML (2009). Case-matched comparison of long-term results of non-heart beating and heart-beating donor renal transplants. Br J Surg.

[5] Hoogland ER, Snoeijs MG, Winkens B, Christaans MH, va Heurn LW (2011). Kidney transplantation from donors after cardiac death: uncontrolled versus controlled donation. Am J Transplant.

[6] Harper SJF, Hosgood SA, Waller HL, Yang B, Kay MD, Gonclaves I (2008). The effect of warm ischaemic time on renal function and injury in the isolated hemoperfused kidney. Transplantation.

[7] Summers DM, Johnson RJ, Hudson A, Collett D, Watson CJ, Bradley JA (2013). Effect of donor age and cold storage time on outcome in recipients of kidneys donated after circulatory death in the UK: a cohort study. Lancet.

[8] Hosgood SA, Nicholson ML (2011) First in man renal transplantation after ex vivo normothermic perfusion. Transplantation 10.1097/TP.0b013e31822d4e04 21841540

[9] Nicholson ML, Hosgood SA (2013). Renal transplantation after ex vivo normothermic perfusion: the first clinical study. Am J Transplant.

[10] Hosgood SA, Patel M, Nicholson ML (2013). The conditioning effect of ex vivo normothermic perfusion in an experimental kidney model. J Surg Res.

[11] Giraud S, Favreau F, Chatauret N, Thuillier R, Maiga S, Hauet T (2011). Contribution of large pig for renal ischemia-reperfusion and transplantation studies: the pre-clinical model. J Biomed Biotechnol.

[12] Hosgood SA, Barlow AD, Yates PJ, Snoesijs MG, van Heurn EL, Nicholson ML (2011). A pilot study assessing the feasibility of a short period of normothermic preservation in an experimental model of non heart beating kidneys. J Surg Res.

[13] Parekh DJ, Weinberg JM, Ercole B, Torkko KC, Hilton W, Bennett M (2013). Tolerance of the human kidney to isolated controlled ischemia. J Am Soc Nephrol.

[14] Alejandro V, Scandling JD, Sibley RK, Dafoe D, Alfrey E, Deen W (1995). Mechanisms of filtration failure during postischemic injury of the human kidney. A study of the reperfused renal allograft. J Clin Invest.

[15] Hosgood SA, Bagul A, Nicholson ML (2011). Minimising cold ischaemic injury in an experimental model of kidney transplantation. Eur J Clin Invest.

[16] Castelao AM, Griñó JM, González C, Franco E, GilVernet S, Andrés E (1993). Update of our experience in long-term renal function of kidneys transplanted from non-heart-beating cadaver donors. Transplant Proc.

[17] Shiroki R, Hoshinaga K, Higuchi T, Tsukiashi Y, Kubota Y, Maruyama T (1998). Prolonged warm ischemia affects long-term prognosis of kidney transplant allografts from non-heart-beating donors. Transplant Proc.

[18] Takai K, Kishi Y, Fujikawa K, Uchiyama K, Tsuchida M, Naito K (2003). Delayed graft function after renal transplantation from a non-heart-beating donors. Transplant Proc.

[19] Yoshida K, Endo T, Saito T, Ikeda M, Kamata K, Baba S (2002). Cadaveric renal transplantation from non-heart-beating donors with graft survival for more than 10 years. Transplant Proc.

[20] Tanabe K, Oshima T, Tokumoto T, Ishikawa N, Kanematsu A, Shinmura H (1998). Long-term renal function in non-heart-beating donor kidney transplantation: a single-center experience. Transplantation.

[21] Reid AW, Harper S, Jackson CH, Wells AC, Summers DM, Gjorgjimajkoska O (2011). Expansion of the kidney donor pool by using cardiac death donors with prolonged time to cardiorespiratory arrest. Am J Transp.

